# The effect of FcRn inhibition on overlapping autoimmune comorbidity in AChR-seropositive generalized myasthenia gravis

**DOI:** 10.3389/fneur.2026.1919993

**Published:** 2026-10-06

**Authors:** Vincenzo Di Stefano, Nicasio Rini, Sofia Campo, Flora D’Amico, Aurora Meli, Simona Maccora, Filippo Brighina, Liliana Bevilacqua, Paolo Barone, Alba Rosa Pati, Mariangela Ferrara, Augusto Maria Rini, Claudia Vinciguerra

**Affiliations:** 1 Department of Biomedicine, Neuroscience and Advanced Diagnostics (BiND), University of Palermo, Palermo, Italy; 2 Neurology Unit, Department of Medicine, Surgery and Dentistry "Scuola Medica Salernitana, Neuroscience Section, University Hospital “San Giovanni di Dio e Ruggi d’Aragona, Salerno, Italy; 3 IRCCS SYNLAB- SDN, Napoli, Italy; 4 Neurology/Stroke Unit, Hospital A. Perrino, Brindisi, Italy

**Keywords:** AChR antibodies, comorbidity, diabetes, Efgartigimod, FcRn inhibition, Lambert-Eaton, myasthenia gravis, stiff-person syndrome

## Abstract

**Introduction:**

Generalized Myasthenia gravis (gMG) is a rare autoimmune disorder of the neuromuscular junction caused by IgG autoantibodies against antigens in the postsynaptic cleft. This study aimed to assess the real-world effectiveness and safety of Efgartigimod (EFG) in AChR-positive gMG with overlapping autoimmune comorbidity.

**Methods:**

EFG was administered as 4 consecutive weekly infusions or injections. Myasthenia Gravis Activity of Daily Living (MG-ADL) and Myasthenia Gravis quantitative (QMG) scales were administered at baseline and after 4 weeks. Clinical data on autoimmune comorbidities and prednisone use were collected.

**Results:**

35 patients (20 women, mean age 53.6 years) received EFG, with a significant and clinical improvement in both MG-ADL and QMG scores. MG-ADL responder rate (MG-ADL reduction≥2) was 79%, while 68% on QMG (QMG reduction≥3); Minimal symptoms expression was reached in 4 patients, with a mean reduction in daily prednisone dose of 8 mg. Overlapping autoimmune comorbidity was reported in 12 gMG patients (34%). gMG patients with Stiff-Person syndrome, type 1 Diabetes and Lambert-Eaton myasthenic syndrome benefited more from EFG with a higher reduction on MG-ADL.

**Conclusion:**

Overlapping autoimmune comorbidity is not rare. EFG demonstrated a meaningful clinical benefit in patients with AChR-positive gMG with overlapping IgG-mediated disorders.

## Introduction

Generalized Myasthenia Gravis (gMG) is a rare autoimmune disease that targets the neuromuscular junction (NMJ), causing fluctuating fatigable weakness of voluntary muscles. In most gMG patients, pathogenetic acetylcholine receptor IgG antibodies (AChR-Ab) interfere with neuromuscular transmission through binding, blocking and/or damage to AChR-receptors. Consequently, patients experience weakness and fatigability affecting ocular, bulbar, respiratory, axial, and limb muscles. A recent population-based study highlighted a rising incidence of autoimmune conditions, now affecting approximately 10% of the global population, with an increasing trend in their co-occurrence posing a substantial socio-economic burden (1). Shared genetic pathways, environmental exposures, and epigenetic mechanisms contribute to the development of overlapping autoimmune syndromes, emphasizing the need for integrated and personalized therapeutic strategies (2, 3). The prevalence of autoimmune comorbidities in MG is estimated at 11–13% (4–6). Autoimmune thyroid diseases are the most frequently co-occurring conditions, followed by systemic lupus erythematosus, rheumatoid arthritis, vitiligo, and type 1 diabetes mellitus (7, 8) (Figure 1A).

**Figure 1:**
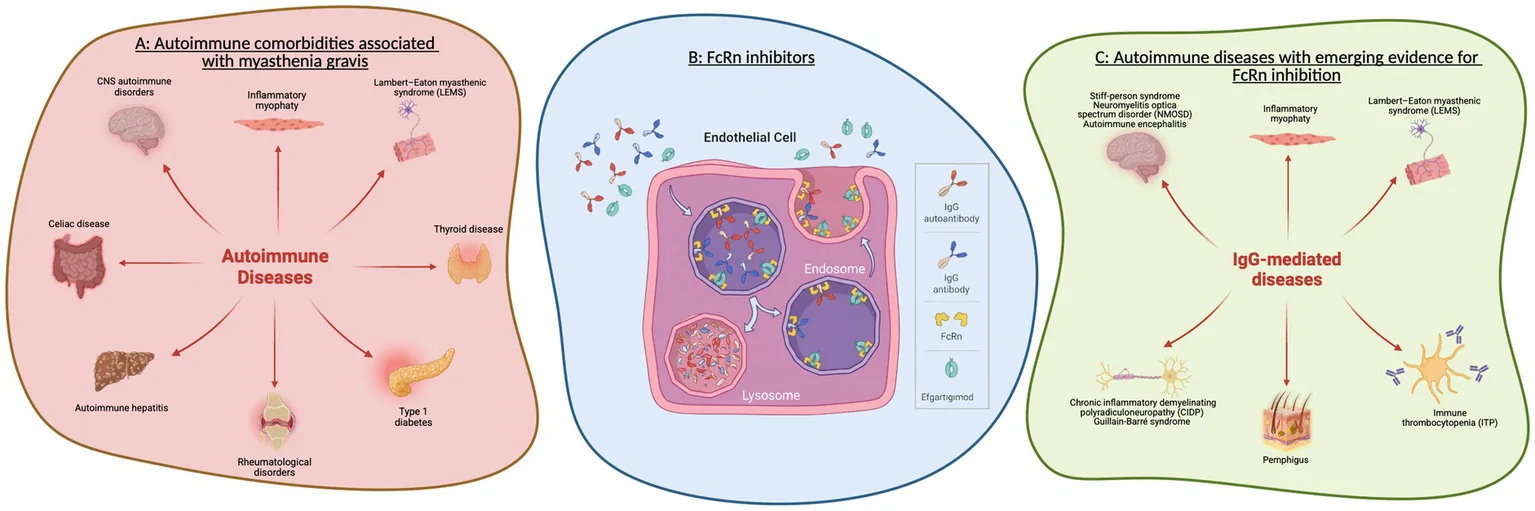
Autoimmune comorbidities and FcRn inhibitor in generalized Myasthenia Gravis (gMG). **(A)** Autoimmune comorbidities associated with gMG. **(B)** Mechanism of action of FcRn inhibitors, showing blockade of FcRn-mediated IgG recycling and enhanced lysosomal degradation of pathogenic IgG. **(C)** IgG-mediated autoimmune diseases with emerging evidence for FcRn inhibitor efficacy (11).

Conventional immunomodulatory therapies such as plasmapheresis (PLEX), immunoadsorption, intravenous immunoglobulins (IVIG), and B-cell depleting agents are employed to reduce circulating pathogenic IgGs. More recently, therapies targeting the neonatal Fc receptor (FcRn) have emerged as a selective strategy to lower IgG levels. FcRn, a heterodimeric receptor expressed on endothelial and other cell types, plays a key role in IgG and albumin recycling, maternal IgG transport, and antigen presentation. Through fluid-phase pinocytosis, IgGs enter endosomes, where FcRn binds them in acidic conditions, preventing lysosomal degradation and allowing their return to the circulation. Thus, serum IgG concentrations are maintained more by FcRn-mediated recycling than by *de novo* IgG synthesis (9) (Figure 1B). Efgartigimod (EFG), a humanized IgG1 Fc fragment engineered via ABDEG technology, has demonstrated a favorable safety and tolerability profile in preclinical and clinical studies (10). It has proven effective in various IgG-mediated diseases, including gMG (both IV and SC formulations) (11–15), immune thrombocytopenic purpura (ITP) (16), and pemphigus vulgaris and foliaceus (with clinical efficacy confirmed for the IV route) (17, 18) (Figure 1C). Subcutaneous efgartigimod has also shown benefit in chronic inflammatory demyelinating polyneuropathy (CIDP) (19) and is under investigation for primary Sjogren’s syndrome. Preliminary studies suggest its potential utility in other neurologic autoimmune diseases such as Guillain-Barré syndrome, autoimmune encephalitis, and neuromyelitis optica spectrum disorders (20–22). Additional FcRn antagonists include rozanolixizumab, batoclimab, and nipocalimab, all of which have shown clinical benefit in gMG (23–25). Among antibody-mediated diseases, rheumatoid arthritis (RA), ITP, pemphigus, MG, and Stiff Person Syndrome (SPS) involve IgG-driven mechanisms, including immune complex deposition and direct binding of autoantibodies to target antigens, leading to tissue damage (26–28). Case reports have suggested that FcRn blockade may be beneficial in patients with multiple IgG-mediated autoimmune disorders (Figure 1C). For instance, EFG treatment led to significant improvement in three patients with concomitant MG and SPS, demonstrating reduced disease burden and a steroid-sparing effect after just two cycles (29). A separate case described by Watanabe et al. reported rapid clinical recovery in a patient with overlapping anti-GQ1b syndrome and MG following EFG therapy (30). Based on these observations, we evaluated the clinical and mechanistic benefits of EFG in MG patients with concurrent autoimmune diseases, aiming to explore the role of FcRn inhibition as a strategic treatment in this complex subset of patients.

## Materials and methods

### Study design and objectives

The safety and efficacy of EFG in gMG patients with overlapping autoimmune comorbidity was investigated in a real-world setting through a multicenter, observational-retrospective study. Patients were enrolled from three Italian tertiary referral centers for Myasthenia Gravis (UOSD Neurofisiopatologia, AUOP Giaccone, Palermo; Neurology Unit, AO Brindisi, Brindisi; Neurology Unit, University Hospital San Giovanni di Dio e Ruggi D’Aragona, Salerno). The principal aim of this study was to investigate the efficacy and safety of EFG in overlapping IgG-mediated autoimmune comorbidity in AChR-seropositive gMG.

### Participants

The following criteria were considered for study inclusion: age ≥ 18 years; informed consent for study participation; Diagnosis of gMG according to standard guidelines and recommendations (31); serum antibody test for Anti-AChR; Treatment with EFG as part of clinical practice (32, 33).

### Procedures

For each recruiting center all patients with acetylcholine receptor antibody-positive (AChR-Ab+) gMG treated with EFG, according to Italian criteria for prescription (MG-ADL score>5 at baseline and received the FcRn inhibitor after initiation of steroids, pyridostigmine and/or immunosuppressants) were enrolled. EFG was administered intravenously at a dosage of 10 mg/kg or subcutaneously at the dose of 1,000 mg, once weekly for four consecutive weeks (11, 13). Patients were stratified at baseline for severity (34), the age at disease onset, gender, comorbidity status and serological status.

### Data collection

Data were retrospectively extracted from clinical records at participating centers over the period January–March 2026. EFG was administered according to routine clinical practice, while a subset of patients accessed treatment through an Expanded Early Access Program for intravenous efgartigimod in gMG (GENERATIVE Protocol). For each patient, demographic and clinical characteristics were collected, including age, sex, body weight, comorbid conditions, age at MG onset, and disease duration at baseline. Disease severity at onset was classified according to the Myasthenia Gravis Foundation of America (MGFA) (34). Information on thymectomy and thymic status was also recorded, along with prior therapeutic history, comprising prednisone, non-steroidal immunosuppressive therapies (NSIST), and maintenance treatment with intravenous immunoglobulin (IVIg) or plasma exchange (PLEX). The number of IVIg or PLEX cycles administered in the year preceding EFG initiation was also documented. During follow-up, any modifications to concomitant treatments, as well as adverse events and reasons for treatment discontinuation, were systematically captured. Serum IgG levels and routine hematological parameters were monitored throughout the treatment period.

### Clinical assessment

Clinical efficacy was evaluated using the Myasthenia Gravis Activities of Daily Living (MG-ADL) scale and the Quantitative Myasthenia Gravis (QMG) score. Clinical measures were recorded at baseline, after the first EFG treatment cycle (week 4), and at the final treatment cycle. For patients who received more than one cycle, clinical measures were collected at baseline and at the end of each cycle. Based on previous evidence, a decrease of at least 2 points in MG-ADL or 3 points in QMG was considered clinically meaningful (11). Minimal symptom expression (MSE) was also assessed and defined as an MG-ADL score of 0 or 1 (35). In patients with overlapping SPS, the severity of SPS syndrome was assessed with the “SPS activity of daily living (SPS-ADL) score” (patient-reported, physician-recorded outcome measure), adapted to identify and measure the impact of SPS symptoms in everyday life (29); the overall disability from MG and SPS was assessed by the modified Rankin scale (mRS).

### Statistical analysis

Categorical variables were summarized as counts and percentages, whereas continuous variables were expressed as mean ± standard deviation (SD). The distribution of MG-ADL and QMG scores was visually inspected using boxplots and formally tested for normality with the Shapiro–Wilk test. In the absence of significant outliers and when normality assumptions were satisfied, longitudinal within-group comparisons were performed using the Mann–Whitney U test. Treatment effectiveness was further evaluated by calculating the change in clinical scores between baseline and week 4 (e.g., ΔMG-ADL = MG-ADLW4 - MG-ADLT0). Associations between continuous variables were explored using Spearman’s rank correlation coefficient. Additional analyses were conducted adjusting for potential confounders, including sex, age at disease onset, thymectomy status, MGFA classification at onset, and comorbidities. A *p*-value <0.05 was considered statistically significant. All analyses were carried out using SPSS version 26.

## Results

### Demographics

We enrolled 35 patients with AChR-Ab+ gMG treated with EFG in the three referral Centers. The cohort consisted of 20 women (57%) and 15 men (43%) with a mean age of 53.6 ± 18.2 years. Five patients (14%) were affected by thymoma, and 32 (91%) presented at least one comorbidity. The main concomitant therapies were pyridostigmine (100%) and prednisone (80%). Most patients were in class III MGFA (34% IIIA, 31% IIIB), 6 patients (17%) were IVB MGFA class, 2 patients were in IVA, and only one patient (3%) was in IIA MGFA class, and 3 patients were in IIB (8%).

### Efficacy and safety

EFG was well-tolerated and effective in managing gMG symptoms as shown by a significant reduction in MG-ADL scores (*F* = 225.67, *p* < 0.001) with an average reduction in MG-ADL score of 4.7 (SD 3.5) and QMG score of 4.8 (SD 4.2), as shown in Figure 2.

**Figure 2:**
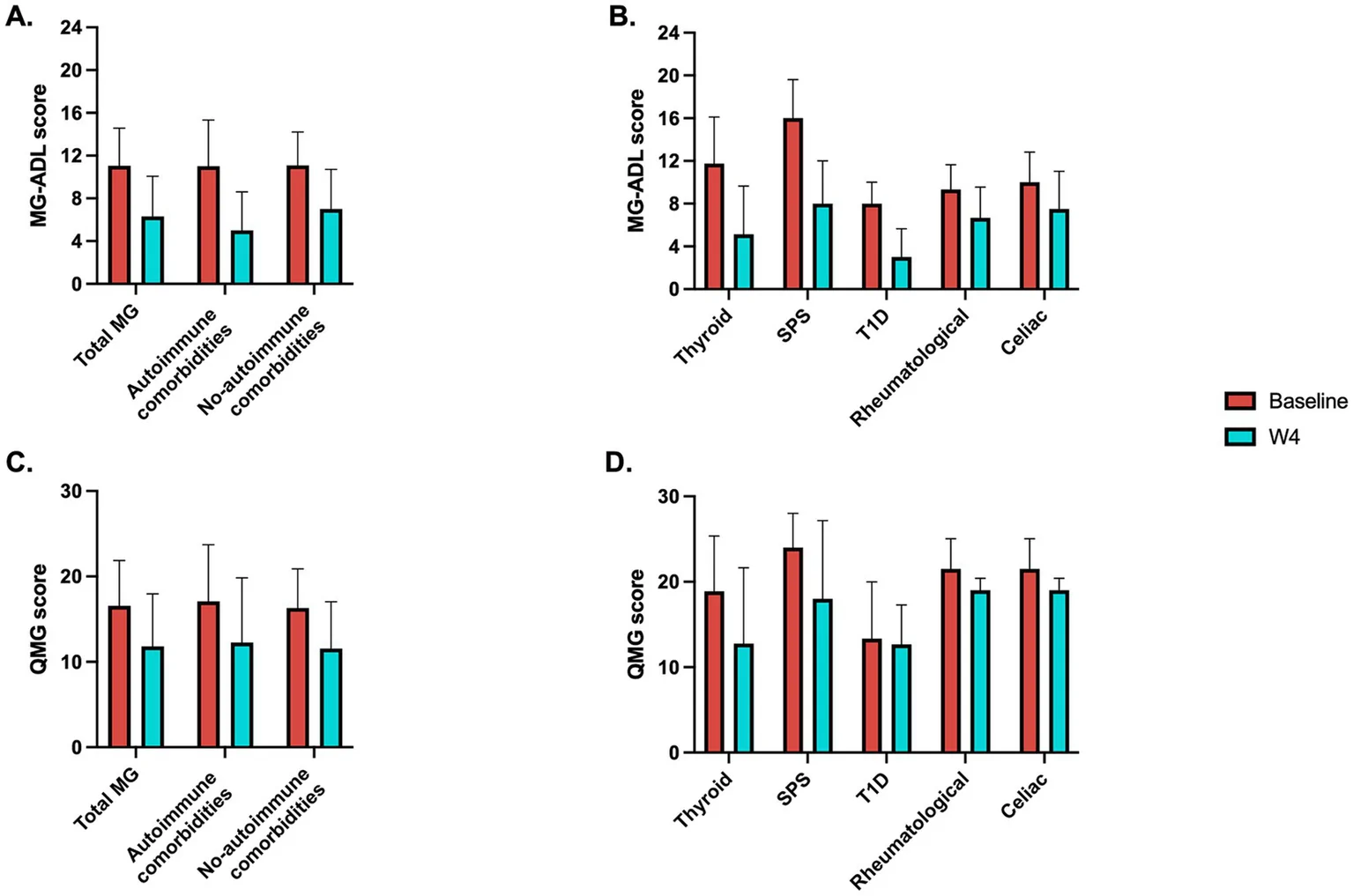
MG-ADL and QMG mean changes at baseline and week 4 during the first cycle of EFG treatment. Top: MG-ADL and QMG scores in the overall cohort **(A,B)**. Bottom: MG-ADL and QMG scores across autoimmune subgroups **(C,D)**. MG-ADL, Myasthenia Gravis Activities of Daily Living scale; QMG, Myasthenia Gravis quantitative scale; MG, Myasthenia Gravis; SPS, Stiff-person syndrome; T1D, Type 1 Diabetes; W, weeks. Error bars represent standard deviation (11).

Responder rate to MG-ADL was 79% while on QMG was 68%. Of interest, 4 patients achieved MSE after the first cycle with EFG (11%). Prednisone was reduced after at least one complete course of treatment with EFG, with an overall mean reduction of about 8 mg at last follow-up available. Also, there was a positive correlation between the MG-ADL score at baseline and ∆MG-ADL (Rho 0.34; *p* = 0.043). Moreover, the entity of reduction of prednisone at the last follow-up was connected to the improvement on MG-ADL (Rho 0.44; *p* = 0.008) and QMG (Rho 0.49; *p* = 0.003) scores. As expected, ∆MG-ADL and ∆QMG were correlated (Rho 0.41; *p* = 0.013). Patients were observed for an average of 382 days (SD 336) and received a mean of 4.17 EFG treatment cycles (SD 3.6). Overall, 30 of 35 patients (85%) did not report any adverse event. The remaining five patients (15%) experienced cutaneous reactions (urticaria and erythema), which led to treatment discontinuation in a single case (case 10).

### Autoimmune comorbidity

Overlapping autoimmune comorbidity was reported in 12 gMG patients (34%). Table 1 reports a comparison between patients with and without autoimmune comorbidity.

**Table 1:** Main baseline demographic and clinical characteristics of patients treated with efgartigimod.

Variable	Autoimmune comorbidity (n. 12, 34%)		gMG without autoimmune comorbidity (n. 23, 66%)		Significance
	Mean/count	SD/%	Mean/count	SD/%	*p*
Age (years)	50.9	18.3	55.0	15.2	0.50
Age at disease onset (years)	41.3	20.0	46.5	18.1	0.38
Gender (men, %)	3	25	12	52	0.12
Body weight (Kg)	68.5	9.7	75.7	17.1	0.28
Disease duration (years)	9.7	10.0	8.7	9.7	0.82
Number of Comorbidities	3.9	4.3	3.9	3.0	0.64
Thymoma (*n*, %)	1	8	4	17	0.43
Concomitant treatments at baseline					
Pyridostigmine (*n*, %)	12	100	23	100	1.0
Prednisone (*n*, %)	7	58	21	91	**0.03**
Azathioprine (*n*, %)	4	33	9	39	0.52
Mycophenolate mofetil (*n*, %)	3	25	7	30	0.53
Methotrexate (*n*, %)	2	17	0	0	0.11
Cyclophosphamide (*n*, %)	1	8	0	0	0.34
Rituximab (*n*, %)	3	25	0	0	**0.034**
C5 inhibitors (*n*, %)	2	17	5	22	0.55
IVIg (*n*, %)	7	58	15	65	0.48
PLEX (*n*, %)	2	17	2	9	0.43
MG subtype (onset)					0.42
Early onset (*n*, %)	8	67	13	57	
Late onset (*n*, %)	4	33	10	43	
MGFA baseline					0.32
IIA (*n*, %)	0	0	1	4	
IIB (*n*, %)	1	8	2	9	
IIIA (*n*, %)	5	42	7	30	
IIIB (*n*, %)	2	17	9	39	
IVA (*n*, %)	2	17	0	0	
IVB (*n*, %)	2	17	4	17	
Serum IgG Baseline (mg/dl)	1001.0	448.3	1010.7	322.4	0.84
MG-ADL Baseline	11.0	4.3	11.1	3.1	0.77
QMG Baseline	17.1	6.6	16.3	4.6	0.74
Prednisone dose at baseline (mg/die)	19.4	27.4	18.7	12.1	0.20
Number of EFG cycles	5.1	4.3	3.7	3.1	0.40
First EFG cycle					
MG-ADL POST (first cycle)	5.0	3.6	7.0	3.7	0.16
∆MG-ADL (first cycle)	6.0	3.7	4.1	3.3	0.38
QMG POST (first cycle)	12.2	7.6	11.6	5.5	0.91
∆QMG (first cycle)	4.8	4.7	4.8	4.0	0.85
Prednisone dose POST first cycle (mg/die)	11.0	14.4	16.6	10.0	**0.04**
Serum IgG POST first cycle (mg/dl)	400.6	110.4	455.3	177.1	0.54
∆IgG first cycle (mg/dl)	600.4	352.4	555.3	209.4	0.97
Last EFG cycle					
MG-ADL PRE (last cycle)	9.9	6.1	9.2	4.3	0.60
MG-ADL POST (last cycle)	6.7	5.7	6.7	4.5	0.88
∆MG-ADL (last cycle)	3.2	3.5	2.5	2.6	0.75
QMG PRE (last cycle)	16.6	7.4	15.0	4.7	0.64
QMG POST (last cycle)	14.5	8.2	12.0	5.8	0.53
∆QMG (last cycle)	2.1	3.4	3.1	4.3	0.46
Prednisone dose after last cycle (mg/die)	8.1	10.2	12.1	9.1	0.17
∆Prednisone last follow-up	11.2	24.2	6.6	9.6	0.91

Continuous variables are expressed as mean with standard deviation (in brackets) and categorical variables as numeric count and percentages. IVIg, Intravenous immunoglobulins; MGFA, Myasthenia Gravis Foundation of America; MG-ADL, Myasthenia Gravis Activity of Daily Living; QMG, Myasthenia Gravis quantitative scale. Bold numbers indicate statistical significance with *p* < 0.05.

The two groups were not different in demographics, disease duration, subtypes, severity of disease, clinical scales, serum IgG levels and average dose of prednisone at the start of EFG. However, the group of patients with autoimmune comorbidity presented some differences in the previous treatments; indeed, a lower frequency in the intake of prednisone as well as the use of methotrexate, cyclophosphamide, and rituximab were reported (Table 1).

Regarding the first EFG cycle, the group of patients with autoimmune comorbidity presented a lower average score on MG-ADL at last infusion (5.0 vs. 7.0) and about 5 mg of prednisone less, in the absence of relevant differences on QMG and IgG levels. However, if we consider the last EFG cycle, the difference in the MG-ADL score disappeared, while maintaining the slight difference in steroid reduction (Table 1).

Overlapping autoimmune comorbidities tended to aggregate in 6 patients: one patient presented 4 overlapping conditions (patient 4) in addition to gMG, while one patient presented 3 autoimmune comorbidities, and 4 patients presented 2 comorbidities. Six patients presented only one comorbidity. Table 2 reports the distribution of comorbidities in this cohort of patients and provides the outcome after EFG treatment.

**Table 2:** Distribution of autoimmune comorbidity and clinical outcomes in 12 patients affected by generalized myasthenia gravis treated with efgartigimod.

Patient number	Autoimmune comorbidity	Impact of EFG	Reported outcomes
1	Stiff person syndrome, Hashimoto’s thyroiditis	Improvement	Subjective improvement, Reduction of therapy
2	Stiff person syndrome, Hashimoto’s thyroiditis	Improvement	Subjective improvement
3	Stiff person syndrome, Graves’ disease	Improvement	Subjective improvement
4	Celiac disease, Sjogren’s disease, systemic lupus erythematosus, Hashimoto’s thyroiditis	Improvement	Subjective improvement
5	Type 1 Diabetes, Hashimoto’s thyroiditis	Improvement	Reduction of therapy, laboratory improvement
6	Type 1 Diabetes, celiac disease, and rheumatoid arthritis	Improvement	Reduction of therapy, laboratory improvement
7	Lambert-Eaton myasthenic syndrome	Improvement	Subjective improvement, Reduction of therapy
8	Hashimoto’s thyroiditis	Improvement	Subjective improvement, Reduction of therapy, laboratory improvement
9	Hashimoto’s thyroiditis	No change	—
10	Type 1 Diabetes	No change	—
11	Hashimoto’s thyroiditis	No change	—
12	Rheumatoid arthritis	No change	—

EFG, efgartigimod.

### Autoimmune thyroid disease

Eight patients of 35 (22.8%) were affected by autoimmune thyroid disease, as a preexisting condition to MG (Cases 1, 2, 3, 4, 5, 8, 9, and 11, Supplementary material). All patients were receiving thyroid hormone replacement therapy for hypothyroidism. One of these patients (case 3) had Graves’ disease treated with antithyroid drugs and was subsequently maintained on levothyroxine replacement, whereas the remaining patients had chronic autoimmune (Hashimoto’s) thyroiditis. In the majority of cases, no clinical or laboratory improvement was observed in the management of the autoimmune comorbidity under investigation. Only 1 out of 8 patients (12.5%), following treatment with EFG, experienced both clinical and laboratory improvement, which led to stabilization of hormone therapy with appropriate adjustment of TSH levels (patient 8 from Supplementary material). This benefit was achieved after a single treatment cycle.

### Stiff-person syndrome

SPS was identified in 3 patients (9%) within our gMG cohort (cases 1–3, Supplementary materials). In all cases, the onset of SPS followed gMG of approximately 1 to 4 years. All three patients had early-onset MG and had undergone thymectomy, with histological evidence of thymic hyperplasia in all cases. At treatment initiation, all patients were receiving ongoing immunotherapy, including corticosteroids and/or conventional immunosuppressants, and had previously been treated with IVIg and, in some cases, PLEX, with incomplete or transient benefit. EFG was therefore introduced as part of a combination therapeutic strategy. Of interest, SPS patients showed higher MG-ADL and QMG scores before EFG initiation (Figures 3A,B), but all patients experienced clinical improvement during treatment cycles, particularly in stiffness, frequency of spasms, and exaggerated startle response, as reflected by changes in clinical outcome measures, with a mean improvement of 8 points in MG-ADL after the first treatment cycle (Figures 2C,D).

**Figure 3:**
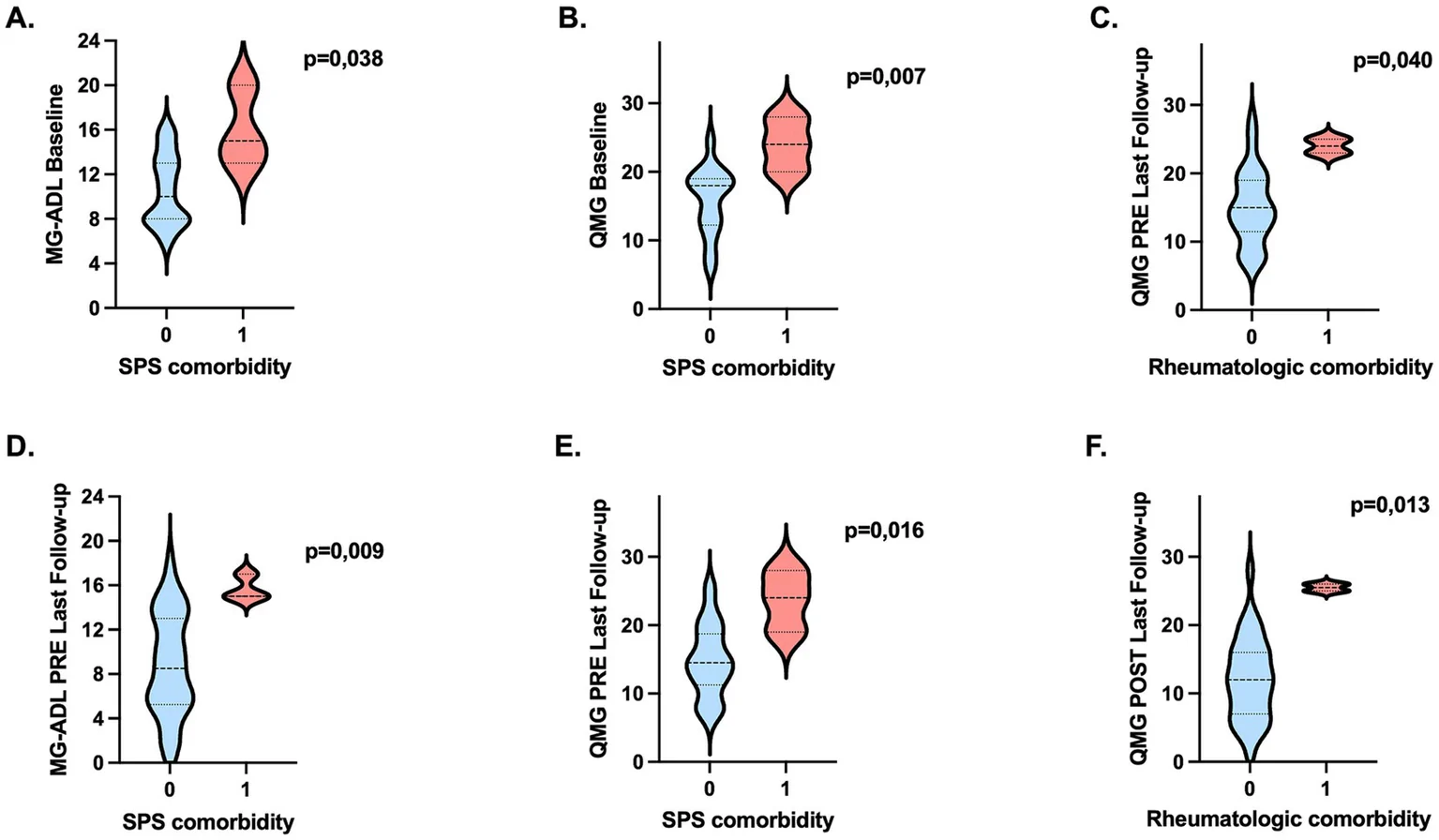
MG-ADL and QMG scores according to SPS and rheumatologic comorbidities. Violin plots show the distribution of MG-ADL and QMG scores in patients with and without SPS **(A–E)** or rheumatologic comorbidities **(C, F)**. MG-ADL, Myasthenia Gravis Activities of Daily Living scale; QMG, Myasthenia Gravis quantitative scale; SPS, Stiff-person syndrome (11).

This aspect was confirmed even in the following cycles, and before receiving the last EFG cycle, SPS patients showed higher MG-ADL and QMG scores again compared to patients without comorbidity (Figures 3C,D). Across the three patients, the SPS-ADL score decreased by an average of 6 points. During EFG treatment, chronic maintenance IVIg was discontinued and the requirement for benzodiazepines to control spasms and stiffness was reduced, although as-needed clonazepam was still required in one patient (case 3) for residual stiff crises. However, the magnitude and durability of response were heterogeneous. Patient 1 showed a marked and sustained improvement, supported by a clinically meaningful reduction in clinical scores. Patient 2 exhibited a partial and time-limited benefit, with improvement during treatment cycles followed by recurrence of symptoms. Patient 3 showed the most pronounced clinical improvement, with a marked overall reduction in symptoms’ severity. A progressive reduction in corticosteroid dosage was achieved in all patients, although complete withdrawal was not possible. In parallel, treatment was associated with a substantial reduction in total IgG levels, with a mean decrease of 651 ± 478 mg/dL. A marked decline in anti-GAD antibody levels was also observed, particularly after the second treatment cycle, with mean values decreasing from 19.4 ± 7.7 to 1.15 ± 0.07 UI/mL. Overall, clinical improvement was supported by both objective measures, including changes in clinical scales, and consistent clinical observations across treatment cycles, indicating a meaningful therapeutic effect despite inter-individual variability in response.

### Type 1 diabetes

Three patients of 35 (8.6%) were affected by type 1 diabetes mellitus (cases 5, 6 and 10 from Supplementary material). The diagnosis of diabetes was made before the diagnosis of myasthenia in cases 6 and 10 and after gMG in case 5. In one patient, after just 4 days of treatment with Efgartigimod, the glycemic profile improved enough to reduce insulin therapy (case 5 from Supplementary material). At the post-treatment follow-up, the anti-GAD 65 antibody level was less than 1 IU/mL (pre-treatment value: 46.9 IU/mL). After only one month from the end of the cycle of EFG, the dosage of prednisone was reduced from 75 mg to 50 mg and at the last follow-up, after 18 months of treatment with EFG, the patient is taking only 5 mg of prednisone along with an SGLT2 inhibitor for diabetes. Another patient affected by diabetes since childhood improved diabetes control after two years of treatment with EFG, with episodes of hypoglycemia, a reduction in night-time insulin dose from 25 to 15 U, and a reduction in mean daily insulin dose (case 6 from Supplementary material). Currently, the patient takes insulin therapy and an SGLT2 inhibitor for diabetes. One patient (case 10) discontinued efgartigimod early due to an adverse event consisting of urticaria.

### Lambert–Eaton myasthenic syndrome

A Lambert–Eaton myasthenic syndrome (LEMS) overlap was documented in one patient (3%), with symptom onset occurring concomitantly with MG (case 7 from Supplementary material). The patient had previously received corticosteroids and azathioprine, with inadequate clinical control, and was treated with EFG within a combination therapeutic strategy. Treatment with EFG resulted in an initial clinical improvement, with a reduction in fatigability and overall neuromuscular symptoms, reflected by a decrease in MG-ADL score from 16 to 5 after the first treatment cycle, suggesting a beneficial effect on both pre- and post-synaptic dysfunction. This improvement was observed during treatment cycles but proved not durable over time, with progressive reduction of response. Clinical benefit was supported by bedside evaluation and patient-reported outcomes, and was accompanied by a steroid-sparing effect, allowing complete discontinuation of corticosteroids (from 15 mg/day). Despite this initial response, clinical benefit was not sustained over time, with marked fluctuations and a progressive reduction in therapeutic durability, reflected by a shortening duration of benefit. This observation is based on a single patient, followed for, after mandatory immunization the patient was switched to the C5 inhibitor zilucoplan, which provided sustained clinical stabilization.

### Rheumatologic comorbidity

In our cohort, 3 out of 35 patients (8.6%) presented with rheumatological comorbidities (cases 4, 6 and 12 from Supplementary material). One patient was affected by SLE, Sjögren’s syndrome (SS). SLE and SS diagnoses preceded that of MG. Treatment with EFG was associated with clinical improvement of SLE, reflected by a reduction in erythrocyte sedimentation rate (ESR) and subjective symptom improvement. Patients with rheumatoid arthritis (cases 6 and 12) did not show clinical benefit following treatment with EFG. Taken together, patients with rheumatological comorbidity tended to display a high number of comorbidities (median 12 vs. 3; *p* = 0.02) and presented higher scores on QMG before EFG cycles (Figures 3E,F) and showed a minimal response in both MG-ADL and QMG scales (Figures 2C,D).

### Celiac disease

Two patients out of 35 (5.7%) were affected by celiac disease, diagnosed before the diagnosis of gMG. The use of EFG did not result in clinical or laboratory improvement of celiac disease in these patients.

## Discussion

In this study, we investigated the impact of EFG on overlapping autoimmune comorbidity in gMG. FcRn inhibition with EFG offers a targeted strategy to rapidly reduce pathogenic IgG autoantibodies involved in disease activity. Several observations exist in the literature on the benefit of FcRn inhibitors in several IgG-mediated disorders, but there is a lack of studies focused on autoimmune comorbidities in patients treated with EFG for gMG, pemphigus or CIDP. With this in mind, the gathered experience of three referral centers for gMG was the starting point for this study. Indeed, it is not uncommon to face gMG patients with autoimmune comorbidity, and the clinician needs to be aware of the potential risks and benefits of FcRn inhibition in this particular setting. On the one hand, several clinicians are timorous to start EFG in these patients because of concomitant treatment with monoclonal antibodies for osteoporosis or rheumatologic disease; another point is the fear of reappearance of autoimmune comorbidity while tapering steroids when myasthenia gravis is better controlled or in remission. Finally, another concern emerges from the correct timing for vaccinations between EFG cycles. Hence, this topic is very interesting and deserves a better characterization and investigation, especially by sharing the experiences obtained treating patients with double or triple IgG-related pathology.

Taken together, patients with overlapping autoimmune comorbidities represented a minority of patients. This may reflect some degree of caution in initiating biologic therapy in more complex patients, particularly given the limited evidence available regarding the use of EFG in this setting. Alternatively, clinicians may preferentially opt for therapies targeting more upstream pathways of the immune response in these patients. A relevant aspect emerging from our analysis concerns the behavior of clinical outcome measures. In the overall cohort, both MG-ADL and QMG scores significantly improved after EFG treatment. However, in the subgroup of patients with overlapping autoimmune comorbidities, we observed a trend toward a greater reduction in MG-ADL compared to QMG, which did not reach statistical significance. This discrepancy may reflect the intrinsic differences between the two scales. MG-ADL is a patient-reported outcome measure, more sensitive to the subjective perception of daily functional improvement, whereas QMG is a physician-assessed scale that captures objective neuromuscular outcomes. In the context of overlapping autoimmune comorbidities, symptoms such as fatigue, pain, or reduced physical function, particularly in rheumatologic or systemic conditions, may influence MG-ADL. This may result in higher MG-ADL scores reflecting aspects of disease burden that are not captured by QMG. Therefore, the combined use of both scales remains essential to obtain a more comprehensive and balanced assessment of treatment response in patients with multiple comorbidities (34, 36).

### Autoimmune thyroid disease

It has been reported in the literature that the prevalence of pre-existing thyroid disorders is higher in patients with MG than in healthy individuals (37). Furthermore, according to other studies, 26.8% of patients with MG present with an autoimmune thyroid comorbidity (38). EFG, through FcRn blockade, accelerates IgG catabolism and reduces circulating IgG levels, including thyroid autoantibodies. However, Hashimoto’s thyroiditis is predominantly a T-cell–mediated disease, with autoantibodies playing a secondary role; therefore, the therapeutic effect of IgG depletion may be limited and likely confined to selected patients with a stronger humoral component. In the single patient with Graves’ disease (case 3), the thyroid disorder was long-standing and had already been treated with antithyroid drugs, leaving residual hypothyroidism managed by hormone replacement; accordingly, no active thyroid autoimmunity was expected to respond to EFG. However, this remains an interesting rationale that is largely theoretical, as also reflected in our case series. In our experience, a meaningful effect on thyroid disorders was not reported.

### Stiff-person syndrome

SPS is a rare neurological comorbidity in MG, typically occurring within the broader context of autoimmune overlap and frequently associated with anti-GAD antibodies. Although epidemiological data are limited, SPS remains an uncommon condition, with an estimated incidence of approximately 1 per million, but it is enriched in patients with coexisting autoimmune diseases, including MG (39, 40). SPS is largely mediated by IgG autoantibodies interfering with GABAergic inhibitory pathways, leading to central hyperexcitability. FcRn inhibition with EFG promotes the degradation of circulating IgG, including anti-GAD antibodies, and has been shown to improve both MG and SPS conditions (29). In our cohort, the observed improvement in stiffness and spasms supports a role of circulating antibodies in driving at least part of the disease phenotype, although incomplete or transient responses suggest additional mechanisms, such as intrathecal antibody production or non-humoral pathways. Moreover, the higher scores at baseline on MG-ADL and QMG in SPS patients, as well as the prompt reduction of scores at W4 suggest that a combined effect of EFG in both gMG and SPS pathophysiology. Further studies are needed in SPS patients without gMG to confirm this observation. Overall, EFG appears to be a plausible and biologically grounded option in MG patients with SPS comorbidity.

### Type 1 diabetes

The prevalence of diabetes in patients affected by MG is 15.6% (41). Inhibition of FcRn by Efgartigimod results in the reduction of all pathogenic IgG, including autoantibodies involved in the pathogenesis of type 1 diabetes (42). Moreover, the reduction in the dose of insulin and hypoglycemia, without modifications on the dose of prednisone experienced in our cohort, suggests a benefit on glycemic metabolism during EFG cycles. However, the effect was only evident during EFG cycles and patients had to return to insulin in the interval among cycles; hence, the long-term effects on glycemic metabolism are not clear. Objective glycemic control was mainly documented as a reduction in insulin requirements, whereas HbA1c was not systematically available across the participating centers. Moreover, the observed glycemic improvement should be interpreted with caution, since concomitant factors, including corticosteroid tapering with resolution of steroid-induced hyperglycemia, introduction of SGLT2 inhibitors, and recovery from acute illness, may have contributed independently of FcRn inhibition. Overall, EFG appears to be an option in MG patients with T1D comorbidity. Further studies are needed in T1D patients without gMG to confirm this observation.

### Lambert-Eaton myasthenic syndrome

LEMS is a rare autoimmune disorder of the presynaptic NMJ, caused by IgG antibodies against voltage-gated calcium channels (VGCC), often associated with paraneoplastic conditions. The coexistence of MG and LEMS is uncommon and has been described as a distinct overlap entity (myasthenia gravis–Lambert–Eaton overlap syndrome, MLOS), with only a limited number of cases reported (43). The rationale for FcRn inhibition lies in the pathogenic role of circulating IgG antibodies targeting VGCC. By accelerating IgG catabolism, EFG may reduce the burden of pathogenic antibodies and improve neuromuscular transmission. Emerging evidence supports this approach, as recent reports have shown that EFG can induce rapid clinical improvement, with concomitant reductions in total IgG and VGCC antibody levels, even in severe or refractory cases (44). In our patient, the initial benefit aligns with these observations; however, the lack of sustained response suggests that additional mechanisms or disease severity may limit long-term efficacy. Overall, EFG represents a good option in MG–LEMS overlap, although its effect may be variable and not consistently durable.

### Rheumatological comorbidity

Systematic review data indicate that the prevalence of rheumatoid arthritis, SLE and Sjögren’s syndrome among MG patients is approximately 3, 2 and 1%, respectively (7). A nationwide Taiwanese study reported a 6.25-fold increased risk of autoimmune rheumatic diseases in MG patients compared to controls. Thymectomy may further increase the risk of SLE, although this remains debated (45). The coexistence of MG and primary Sjogren’s syndrome is rare but documented, with a systematic review identifying 17 cases (46). In AChR-positive MG, autoantibodies are mainly IgG1 and IgG3, which fix complement and damage the NMJ. A similar pattern is observed in SLE, where IgG1/3 autoantibodies drive immune-complex deposition and complement-mediated organ damage; Sjogren’s syndrome also involves IgG-mediated glandular injury (anti-SSA/Ro, anti-SSB/La). In rheumatoid arthritis, autoantibodies (e.g., ACPA, predominantly IgG1) contribute to synovial inflammation but mainly alongside T-cell and cytokine pathways. By reducing circulating IgG, FcRn inhibition is expected to attenuate SLE activity and potentially Sjogren’s syndrome. In rheumatoid arthritis, although IgG autoantibodies may decrease, the clinical impact may be limited without targeting T-cell and cytokine-driven mechanisms. Our experience suggests that patients with rheumatologic comorbidity are more complex and severe, displaying higher scores on QMG and often present a reduced response to EFG. This piece of evidence does not support an effect of EFG on this kind of comorbidity. On the contrary, there is a possibility that in these patients the entity of gMG might be overestimated on MG-ADL due to the overlap among symptoms of myasthenia and rheumatologic diseases, thus explaining a reduced response to treatments. However, the limited clinical response observed in patients with rheumatological comorbidities may not be attributable solely to the complexity or severity of these disorders, but could also reflect their distinct immunopathogenic mechanisms, which may be less amenable to the therapeutic effects of EFG.

### Celiac disease

The co-occurrence of celiac disease and MG is possible, but it is rare and not supported by strong epidemiological evidence of an association in the literature. In celiac disease, the destruction of the intestinal mucosa is mediated mainly by T lymphocytes. Furthermore, the subsequent activation of B lymphocytes leads to the production of autoantibodies not only belonging to the IgG class but also, and especially, of the IgA type (47). This mechanism could be a reason for the lack of effect of EFG on celiac disease, considering the selective role in the reduction of pathogenic IgG autoantibodies.

## Limitations

Several limitations should be acknowledged, including the potential confounding effect of concomitant immunosuppressive therapies, the small sample size within individual disease subgroups, and the heterogeneity of the autoimmune conditions included in the analysis substantially limits the statistical power of the study and prevents definitive conclusions regarding the efficacy of efgartigimod on specific autoimmune disorders. The findings observed in patients with stiff-person syndrome, type 1 diabetes, and Lambert-Eaton myasthenic syndrome should therefore be considered exploratory and hypothesis-generating. Moreover, the retrospective observational design and the real-world setting may have introduced selection and information biases, including heterogeneity in treatment decisions, concomitant therapies, follow-up duration, and timing of clinical assessments across centers. Also, the absence of a matched control group precludes distinguishing the effects of efgartigimod from spontaneous fluctuations in disease activity or from the effects of concomitant immunotherapies and corticosteroid adjustments. This is particularly relevant when assessing the impact of treatment on autoimmune comorbidities, for which disease-specific clinical and laboratory outcomes were not systematically available for all patients. In addition, the use of MG-ADL in patients with overlapping systemic or rheumatological diseases may be influenced by symptoms such as fatigue, pain, and functional impairment that are not exclusively attributable to myasthenia gravis. In addition, disease-specific quality-of-life measures were not systematically collected, particularly in patients with rheumatologic or systemic comorbidities, which limits the assessment of the subjective burden of overlapping conditions. Furthermore, a further bias comes from the mixed use of intravenous and subcutaneous formulations to treat MG patients with autoimmune comorbidity. Also, due to the multicentric nature of the study, in most cases the laboratory improvement was subjective as different clinicians evaluated results from different laboratories; regarding the diagnosis of autoimmune thyroiditis, it was collected retrospectively based on endocrinological evaluation prior to the start of EFG, but we are aware that high heterogeneity was present among different centers. Regarding SPS, the improvement was directly reported by the patients but was also demonstrated by improvement on modified rankin scale and SPS-ADL, but a validated scale specific for SPS is still on demand. Hence, for these aspects our results should be taken cautiously. Finally, although the mean follow-up was approximately one year, the duration of treatment and observation was heterogeneous, limiting the assessment of the long-term durability of response and safety. Prospective, adequately powered studies with standardized disease-specific outcome measures, longer follow-up, and appropriate control groups are warranted to confirm these preliminary observations.

## Conclusion

In this multicenter real-world analysis, efgartigimod was associated with a rapid and clinically meaningful improvement in patients with generalized myasthenia gravis. Significant decreases in MG-ADL and QMG scores were evident after a single treatment cycle, along with high response rates and a favorable safety profile, irrespective of the presence of autoimmune comorbidities. Continued administration in most patients enabled sustained clinical benefit over time. Notably, the treatment was well tolerated even among individuals with severe disease and a substantial comorbidity burden, and in some cases facilitated a reduction in concomitant corticosteroid therapy. When considering overlapping autoimmune comorbidity meaningful improvements were observed in patients with stiff-person syndrome, type 1 diabetes, and Lambert–Eaton myasthenic syndrome, whereas no significant impact was detected in celiac disease, thyroid disorders, or rheumatologic conditions. Although the findings are limited by the small sample size and retrospective design, they potentially suggest that efgartigimod may represent a promising therapeutic option for patients with coexisting autoimmune diseases. Further prospective studies with larger cohorts and longer follow-up are needed to validate these results and to better define the role of efgartigimod in IgG-mediated disorders.

## Data Availability

The raw data supporting the conclusions of this article will be made available by the authors, without undue reservation.
