# Supplementary material for: The effect of FcRn inhibition on overlapping autoimmune comorbidity in AChR-seropositive generalized myasthenia gravis
Source: Front Neurol. 2026 Oct 6;17:1919993. doi: 10.3389/fneur.2026.1919993 (PMC13680258; doi:10.3389/fneur.2026.1919993)
Supplement: Supplementary file 1 [file Supplementary_file_1.DOCX]

**Supplementary material**

**Case 1**

A 34-year-old woman was referred in 2021 for a rapidly progressive generalized myasthenia gravis (MG), presenting with prominent fatigability, proximal limb weakness, impaired arm elevation, dysphagia, chewing difficulties, fluctuating ptosis, and evening diplopia (MGFA class IVA; MG-ADL 20). Her past medical history included autoimmune thyroiditis, bronchial asthma, and anxiety-depressive disorder, suggesting an underlying susceptibility to immune-mediated conditions. Initial management consisted of intravenous immunoglobulin (IVIg) as rescue therapy and high-dose corticosteroids, which resulted in only partial symptom control. Azathioprine was subsequently introduced but discontinued due to hepatotoxicity. Approximately one year after MG onset, the patient developed additional neurological manifestations, including impaired attention and memory, exaggerated startle response, gait instability with recurrent falls, diffuse pain, and episodic muscle spasms. Further investigations demonstrated elevated anti-glutamic acid decarboxylase (GAD) antibodies and continuous motor unit activity on electromyography, supporting the diagnosis of stiff-person syndrome (SPS), thus defining an overlap of antibody-mediated neurological disorders. Given the inadequate response to conventional immunotherapies and the shared IgG-driven pathogenesis of both conditions, treatment with EFG was initiated. Following therapy, the patient exhibited a pronounced clinical improvement involving both neuromuscular junction and central hyperexcitability symptoms. Spasms and startle response resolved completely, while rigidity significantly decreased, reflecting a global improvement of disease burden. A substantial reduction in SPS-ADL (-9 points) and MG-ADL (-12 points, 20 at baseline vs 8 after first cycle of EFG) scores was observed. In parallel, a decrease in circulating pathogenic antibodies was observed, with reduction of both anti-AChR and anti-GAD titers, consistent with the pharmacodynamic effect of FcRn inhibition. During follow-up, which lasted approximately three years, the therapeutic response was maintained across subsequent treatment cycles, with sustained clinical response and no requirement for rescue therapies. During EFG treatment, chronic maintenance IVIg was discontinued and the need for benzodiazepines to control spasms and stiffness was reduced. Notably, corticosteroid dosage was progressively reduced, from 20 mg daily to 10 mg at last follow up, supporting a clinically meaningful steroid-sparing effect. EFG was initially administered intravenously at a dose of 10 mg/kg per infusion. During follow-up, treatment was transitioned to the subcutaneous formulation (1000 mg per administration), after which a mild local injection-site reaction characterized by erythema was reported, without the need for treatment discontinuation. No other relevant adverse events were observed. At the last follow-up, the patient remained clinically improved, reporting increased muscle strength and reduced fatigability, along with decreased rigidity and fewer paroxysmal spasms. MG-ADL and QMG further improved compared to baseline (-7 and -8 points, respectively), confirming a sustained benefit over time.

**Case 2**

A 49-year-old woman was diagnosed with AChR-positive generalized myasthenia gravis (MG) after presenting with diffuse weakness, marked fatigue, ptosis, and diplopia (MGFA class IIA; MG-ADL 9). Her medical history was significant for autoimmune thyroiditis and mood disorder. The patient initially showed a favorable response to intravenous immunoglobulin (IVIg) and was subsequently treated with prednisone and azathioprine for maintenance. Four years after MG onset, she developed new neurological symptoms, including cognitive dysfunction, progressive gait impairment with recurrent falls, and widespread pain. Neuroimaging did not reveal structural abnormalities. Over time, the clinical course progressively evolved with the appearance of painful spasms, exaggerated startle response, axial stiffness, dysarthria, dyspnea, and dysphagia. In light of the coexistence of multiple autoimmune conditions, stiff-person syndrome (SPS) was suspected and later confirmed by the detection of anti-GAD antibodies in serum and cerebrospinal fluid, along with supportive neurophysiological findings. Despite treatment with IVIg (2 g/kg over 5 days), no significant benefit was achieved in controlling SPS symptoms, and the disease course was characterized by recurrent exacerbations of MG and SPS, several hospitalizations and progressive disability. Considering the overlapping IgG-mediated mechanisms and the insufficient response to standard therapies, several advanced treatment strategies were explored, including plasma exchange (5 cycles), cyclophosphamide 1 gr (5 administrations), complement inhibition with ravulizumab (3300 mg per infusion, administered every 8 weeks; three infusions in total), and FcRn inhibition with EFG. EFG therapy was associated with an initial clinical improvement, with a reduction in MG-ADL (15 at baseline vs 12 after first cycle of EFG) and a concomitant improvement in SPS manifestations, including decreased leg stiffness and reduced frequency of spasms and exaggerated startle response, accompanied by a reduction in SPS-ADL score. However, this effect was not sustained over time, and the patient experienced subsequent relapses with persistent severe SPS symptoms, including frequent and prolonged stiff crises. Given the incomplete and transient response to FcRn inhibition, escalation to rituximab was undertaken, resulting in a reduction in spasm frequency and improvement in motor control.

**Case 3**

A 61-year-old woman who was admitted to Neurology ward several times for diplopia, ptosis, dyspnea, dysphagia, and fatigue, but due to seronegative AChR-Ab testing was initially diagnosed as affected by functional neurological disorder. Her medical history mentioned only Graves’ disease. Some clinicians hypothesized a seronegative MG, and she was treated with pyridostigmine and prednisone with a good response on ptosis and diplopia. Due to a finding of thymic residual, she underwent thymectomy in 2019 and was treated with IVIg with clinical benefit. After reduction of the dosage of prednisone, anti-AChR-Abs were detected in three different samples, as well as a neurophysiological confirmation of MG diagnosis was obtained with SFEMG (MGFA IIb). However, after a couple of years she complained of balance difficulties with frequent falls and cognitive impairment associated with spasms. Clinical and neurophysiological examination showed the presence of an exaggerated startle response, increased muscle tone with brisk reflexes, and ataxia. MRI of the brain was unremarkable. Hence, a diagnosis of SPS was confirmed with seropositive anti-GAD Abs in the serum. After a significant response achieved with two cycles of IVIg, she did not benefit from further IVIg cycles. Azathioprine was then started, but it was stopped afterwards for nausea and vomiting. Even Rituximab 1000 mg was started with unsatisfactory response after 6 months. In May 2023, Efgartigimod treatment was started with clinical benefit on myasthenia (MG-ADL 13 at baseline vs MG-ADL 5 after first cycle of EFG) and SPS symptoms. The patient received a total of 14 cycles of drug, of which 3 was administered subcutaneously, without adverse events. Currently, the patient has a clinical benefit that has been maintained over time, after more than three years of treatment, assessed using the MG-ADL and QMG scale; in addition, the patient reports an improvement in stiffness although several stiffness crises persist (about 4 per week) which are effectively treated with clonazepam as needed.

**Case 4**

A 69-year-old woman with a 34-year history of AChR-Ab positive generalized Myasthenia Gravis presented with progressive worsening of symptoms over the previous year. Her medical history was significant for multiple autoimmune diseases, including Systemic Lupus Erythematosus (SLE), Sjögren’s Syndrome, Celiac Disease, Hashimoto’s Thyroiditis and Lymphocytic Colitis. She also suffered from several non-autoimmune conditions, including headache, osteoporosis and restrictive respiratory syndrome. Her previous therapies were Prednisone (discontinued due to bone-related adverse effects), two years of Rituximab, plasmapheresis (discontinued due to vascular access complications) and Azathioprine (discontinued due to headache). At that time, her ongoing therapy consisted of Pyridostigmine 60 mg four times daily, Levothyroxine and Romosozumab administered every six months for osteoporosis. As she was experiencing a relapse, intravenous Efgartigimod was initiated; the MG-ADL score improved from 18 to 5 after two cycles. However, after switching to the subcutaneous formulation, symptoms worsened with the MG-ADL increasing to 14, although she described a subjective improvement in her lupus symptoms (accompanied by a significant reduction in ESR). During this period, the patient also reported widespread pain and a rheumatologic evaluation revealed tenderness at all tender points consistent with Fibromyalgia. Her therapy was therefore intensified by adding Pyridostigmine 180 mg MR at night and then Mycophenolate mofetil 500 mg twice daily. A new cycle of subcutaneous Efgartigimod was attempted; however, no clinical improvement was observed (MG-ADL 18). Consequently, Efgartigimod was discontinued and alternative therapeutic options including intravenous immunoglobulins (IVIG), Cyclophosphamide or Ravulizumab, are currently being evaluated.

**Case 5**

A 77-year-old man was admitted to the emergency department with progressive respiratory problems, difficulty standing, and a fall in August 2024. The patient had AChR-positive generalized myasthenia gravis (Anti-AChR Ab >20 nmol/L), which began in January 2024 with ptosis and diplopia, accompanied by difficulty chewing in the absence of swallowing difficulties (especially with solid foods) and shortness of breath (especially when lying flat). The symptoms were most evident in the morning upon awakening (12 hours after the last dose of pyridostigmine) with slight improvement after starting treatment. His medical history included bronchial asthma, sigmoid diverticulosis, bilateral hepatic and renal cysts, arterial hypertension, cervical disc disease, and diabetes. The patient was then admitted to hospital and treated with intravenous immunoglobulin, with only partial clinical benefit. The patient worsened and was treated with NIV, but repeated trials failed to wean him. He also presented uncontrolled glycemia. At admission HBa1C was 6.3%. The introduction of prednisone 75 mg was unsuccessful; therefore, he began treatment with Efgartigimod, with significant improvement. After just 3 days, the MGADL score had dropped to 6 (12 at admission). The patient was weaned from oxygen therapy, and his glycemic profile improved enough to reduce insulin therapy the following day (Supplementary table 1). Following the patient clinical stabilization, he was discharged home, continuing the infusion of the biologic drug according to protocol under a DH regimen. At the post-treatment follow-up, the patient had MG-ADL 1 and the anti-GAD 65 antibody level was less than 1 IU/mL (pre-treatment value: 46.9 IU/mL). Hence, predisone was progressively reduced to 62.5 mg for two weeks and than reduced to 10 mg in 6 months. Today the patient is undergoing therapy with prednisone, azathioprine, pyridostigmine and is still in minimal symptoms expression without corticosteroids.

|  | **Morning glycemia (mg)/insulin (UI)** | **Afternoon glycemia (mg)/insulin (UI)** | **Evening glycemia (mg)/insulin (UI)** | **Prednisone (mg)** |
| --- | --- | --- | --- | --- |
| 14 August 2026 | - | - | 181 | 50 |
| 15 August 2026 | 136 | - | - | 50 |
| 17 August 2026 | - | 183 (+5 U) | 230 (+10 U) | 50 |
| 18 August 2026 | 128 (no insulin) | 166 (+5 U) | 180 (+10 U, +5 U) | 50 |
| 19 August 2026 | 112 (+5 U) | - (+5 U) | (+10 U, +5 U) | 50 |
| 20 August 2026 | 164 (+5 U) | 241 (+5 U) | 155 (+10 U, +5 U) | 75 |
| 21 August 2026 | 181(+5 U) | 163(+5 U) | 110 (+10 U, +5 U) | 75 |
| 22 August 2026 | 150 (+5 U) | 185(+5 U) | 157(+10 U, +5 U) | 75 |
| 23 August 2026 | 187(+5 U) | 221(+5 U) | 219(+10 U, +5 U) | 75 |
| 24 August 2026 | (+5 U) | (+5 U) | 205(+10 U, +5 U) | 75 |
| 25 August 2026 | 168 (+5 U) | 209 (+5 U) | 166(+10 U, +5 U) | 75 |
| 26 August 2026  (Efgartigimod iv 700mg) | 210 (no insulin) | (+5 U) | 181(+10 U, +5 U) | 75 |
| 27 August 2026 | 102 (no insulin) | 177(+5 U) | 169 (+10 U, +5 U) | 75 |
| 28 August 2026 | 100 (no insulin) | 271 (+5 U) | 230 (+10 U, +5 U) | 75 |
| 29 August 2026 | 128(+5 U) | 219 (+5 U) | 227(+10 U, +5 U) | 75 |
| 30 August 2026 | - (+5 U) | 190 (+5 U) | 144 (+10 U, +5 U) | 75 |
| 31 August 2026 | 146  (+5 U) | 161 (+5 U) | 145 (+10 U, +5 U) | 75 |
| 1 September 2026 | 87 (no insulin) | 157 (+5 U) | 110 (+10 U, +5 U) | 75 |
| 2 September 2026 | 84 (no insulin) | (+5 U) | (+10 U) | 75 |

**Supplementary table 1.** Glycemic profile of patient 5 during hospitalization and after the first week from the treatment with Efgartigimod.

**Case 6**

A 25-year-old man was hospitalized for myasthenic crisis and symptoms such as dysphagia, diplopia, dyspnea, dysphonia and constant fatigue in 2023. The diagnosis of anti-AchR-positive generalized myasthenia gravis was made in 2019 following a history of easy fatigue, ptosis, diplopia, and dyspnea that progressively worsened over the years. The patient was treated with pyridostigmine and prednisone after diagnosis, and following the detection of thymic remnants on a chest CT scan, he underwent thymectomy in 2020 and a repeat operation the following year. His medical history includes arterial hypertension, severe allergic bronchial asthma, celiac disease, type 1 diabetes mellitus, rheumatoid arthritis, heart failure (NYHA class II), mixed dyslipidemia, hyperuricemia, erosive gastropathy with hiatal hernia, colon-sigmoid diverticulosis, congenital hearing loss in the right ear, and gallstones. During hospitalization the patient was treated with intravenous immunoglobulin with clinical benefit. At the post-treatment follow-up, the patient had an MG-ADL of 13, in May 2023, he began therapy with Efgartigimod, with clinical benefit (MG-ADL 8 at baseline vs MG-ADL 5 after first cycle of EFG). After two years of treatment, diabetes control improved with episodes of hypoglycemia, a reduction in nighttime insulin dose from 25 to 15 U, and a reduction in mean daily insulin dose. The drug had no effect on his celiac disease. The patient, after three years of therapy with EFG, is currently still undergoing subcutaneous therapy with clinical benefit (last MG-ADL 4) and without adverse events.

**Case 7**

A 62-year-old female with hypertension, type II diabetes, and dilated cardiomyopathy presented with lower limb weakness and diplopia post-COVID-19 vaccination. Initial findings of low-amplitude CMAPs and albumin-cytological dissociation suggested atypical CIDP. However, the emergence of fluctuating diplopia and steppage gait prompted reassessment. Neurophysiology revealed CMAP decrement at low-frequency RNS with marked post-exercise facilitation; serology confirmed positivity for both anti-AChR and N-type VGCC antibodies. Diagnosed with Myasthenia Gravis-Lambert Eaton Overlap Syndrome (MLOS), management was limited by steroid-induced hyperglycemia and azathioprine-induced pancreatitis. Given the IgG-mediated nature of MLOS, the FcRn antagonist efgartigimod was initiated to broadly deplete the pathogenic autoantibody pool targeting both pre- and post-synaptic sites. Although efgartigimod initially provided clinical relief, its therapeutic durability progressively declined, with a shortening duration of benefit. Following mandatory immunization, the patient transitioned to the C5 inhibitor zilucoplan. This switch achieved rapid and sustained clinical stability. This case underscores that while FcRn-mediated IgG clearance is a rational approach for complex overlap syndromes, C5 inhibition may provide superior long-term stabilization in refractory MLOS cases where antibody-mediated complement activation remains the dominant driver.

**Case 8**

A 33-year-old male presented with AChR-positive generalized Myasthenia Gravis (gMG) and comorbid autoimmune thyroiditis and schizoaffective disorder. The clinical phenotype was severe, featuring bulbar and axial involvement (MGFA IVb). Despite thymectomy for B2 thymoma and conventional immunotherapy, the patient remained refractory. Prednisone (25 mg/day) management was severely constrained by the risk of psychiatric exacerbations, and maintenance PLEX/IVIg failed to prevent recurrent hospitalizations for respiratory deterioration. In February 2025, a single four-week cycle of efgartigimod was initiated. Given the dual IgG-mediated nature of his conditions, efgartigimod was utilized to target the global pathogenic IgG pool. This intervention achieved a profound clinical response (MG-ADL=0). Notably, the targeted reduction of circulating IgG via neonatal Fc receptor (FcRn) antagonism appeared to stabilize the autoimmune multi-organ burden, potentially mitigating the inflammatory drive of the thyroiditis alongside the gMG. At one-year follow-up, the patient maintains sustained Minimal Symptom Expression (MSE) and MG-ADL=0. This enduring stability suggests that FcRn-mediated IgG depletion provides a potent, multi-target modulatory effect, facilitating a successful corticosteroid-sparing strategy and significantly improving both neurological and psychiatric outcomes.

**Case 9**

A 52-year-old woman was affected by early-onset generalized myasthenia gravis and Hashimoto’s thyroiditis. No clinical or laboratory improvement in the autoimmune comorbidity could be detected, as she had undergone thyroidectomy following a diagnosis of papillary thyroid carcinoma and was subsequently maintained on hormone replacement therapy. Regarding myasthenia gravis, she experienced a substantial benefit from EFG, as demonstrated by the MG-ADL and QMG scores (10 and 18 at baseline, respectively, and 5 and 14 after the first cycle of EFG, respectively).

**Case 10**

A 25-year-old woman was affected by generalized myasthenia gravis and type 1 diabetes mellitus, diagnosed approximately ten years earlier. She had poor glycemic control, as demonstrated by persistently elevated glycated hemoglobin levels despite ongoing insulin therapy; it was therefore difficult to determine whether EFG had any impact on this comorbidity. During treatment, she developed cutaneous adverse reactions consisting of erythema and urticaria, which led to discontinuation of EFG.

**Case 11**

A 40-year-old woman had a history of Hashimoto’s thyroiditis, treated with levothyroxine replacement therapy at a dose of 11.5 μg/day. She was diagnosed with generalized acetylcholine receptor antibody-positive (AChR+) myasthenia gravis (MG) in 2002, at the age of 16 years. In 2003, she underwent prophylactic thymectomy for thymic hyperplasia. Over the following years, she was treated with corticosteroids at doses of up to 50 mg/day, which were subsequently tapered and eventually discontinued because of poor tolerability. Azathioprine at a dose of 100 mg/day was also administered and later discontinued because of limited efficacy. In February 2024, she was referred to our center and started treatment with mycophenolate mofetil, titrated to 1,500 mg/day. This resulted in partial disease control, although occasional episodes of dyspnea, binocular diplopia, and fatigability of all four limbs persisted. Therefore, in January 2026, subcutaneous efgartigimod therapy was initiated, resulting in clinical improvement (MG-ADL from 6 to 2 after the first cycle). No changes in thyroid hormone levels or levothyroxine replacement therapy were observed during treatment.

**Case 12**

A 73-year-old woman had a 30-year history of rheumatoid arthritis (RA), previously treated with methotrexate and corticosteroids until 2013, when both were discontinued because of limited clinical benefit. Over the subsequent years, she developed progressive joint deformities and multifocal ankylosis, resulting in marked functional impairment, including limited arm abduction and hand mobility, as well as the need for support when walking. Generalized AChR+ Myasthenia gravis (MG) developed in 2024, initially presenting with fluctuating binocular diplopia. Over the following year, she developed progressively worsening bulbar symptoms, culminating in a myasthenic crisis in June 2025. She was treated with plasma exchange (PLEX), with significant clinical improvement. Afterwards, corticosteroid therapy was progressively tapered; however, treatment was complicated by an L3 vertebral compression fracture. Mycophenolate mofetil was subsequently introduced, with appropriate laboratory monitoring, and titrated to 1,000 mg/day. Because of poor gastrointestinal tolerability, characterized by abdominal cramps and diarrhea, the dose was reduced to 500 mg/day. In January 2026, subcutaneous Efgartigimod was initiated, resulting in further clinical improvement and sustained long-term disease stability (MG-ADL from 8 to 5 after the first cycle). No clinically or laboratory-detectable changes in the underlying rheumatologic disease were observed during treatment.
